# Extremely Rare Primary Spinal Epidural Indolent Mantle Cell Lymphoma: A Case Report With Literature Review

**DOI:** 10.7759/cureus.14762

**Published:** 2021-04-29

**Authors:** Rohit Prasad, Nishan B Pokhrel, Sushil Paudel, Dinesh Kafle, Rohit K Pokharel

**Affiliations:** 1 Orthopaedics, Tribhuvan University Institute of Medicine, Kathmandu, NPL

**Keywords:** indolent, mantle cell, non-hodgkin’s lymphoma, primary spinal epidural lymphoma

## Abstract

Lymphomas are malignant tumors arising from lymphoid tissues and can spread to other organs. Primary extra-nodal locations such as the spinal epidural space are less common. Primary spinal epidural lymphoma (PSEL), which can be either Hodgkin’s or non-Hodgkin’s type, is rare. There are different cell types of Non-Hodgkin’s PSEL, among which mantle cell lymphoma (MCL) is extremely rare. MCL can have an aggressive or indolent course. Indolent MCL in the epidural space is not yet reported. We report a case of 20-year-old male who presented with radiating low back pain for six weeks followed by a progressive neurological deficit in both lower limbs for nine days. Magnetic resonance imaging (MRI) revealed spinal epidural tumor extending from L2 to L3. Decompression and subtotal excision biopsy were performed. Histopathology and immunohistochemistry identified indolent MCL. His neurological status improved to normal postoperatively, and he was referred to an oncologist. He is under observation and planned for radiotherapy. At one-year follow-up, he is asymptomatic and doing his regular job abroad.

## Introduction

Lymphoma, a neoplastic disorder of lymphoid tissue, can involve the vertebral body, epidural compartment, spinal cord, and cauda equina [1]. Primary epidural location of lymphomas, both Hodgkin’s and non-Hodgkin’s, is very rare due to which the diagnosis is challenging. Non-Hodgkin's lymphoma (NHL) compromises different cellular types of lymphomas. Mantle cell lymphoma (MCL) is a mature B-cell NHL [2]. It is characterized by monotonous proliferation of atypical small lymphoid cells growing in mantle zone, nodular, or diffuse patterns [3]. Chromosomal translocation t(11:14) that leads to cell cycle dysregulation is the molecular hallmark [4]. The clinical course of MCL may be aggressive or slow-growing (indolent), which is determined by over- or under-expression of genes such as *SOXII* and *TP53* [5,6]. Mantle Cell Lymphoma International Prognostic Index (MIPI), together with Ki67 protein level, can predict the prognosis of MCL [7].

Primary MCL in the spine and epidural space is extremely rare. To date, there are only three reported cases of primary spinal epidural MCL [8,9]. To the best of our knowledge, this is the first case report on primary spinal epidural indolent mantle cell lymphoma (IMCL) in a young male. This report describes the clinical features, imaging characteristics, histopathological features, immunophenotype, and clinical course of IMCL.

## Case presentation

A 20-year-old immunocompetent male arrived to our emergency department with a chief complaint of low back pain for six weeks followed by radicular pain and difficulty in bearing weight in both lower limbs for nine days. He also complained of decreased sensation over bilateral lower limbs and had normal bowel and bladder habits. He had a road traffic accident six weeks back while riding a motorcycle with minor injuries. There was no history of fever, headache, or night sweats.

On examination, no obvious spinal deformity was appreciated. Localized tenderness was present over the L3/L4 vertebra. Motor power varied across muscle groups: hip flexors (5/5 bilaterally), knee extensors (5/5 bilaterally), ankle dorsiflexors (1/5 on the right, 3/5 on the left), long toe extensors (1/5 on the right, 3/5 on the left), and ankle plantar flexors (3/5 on the right, 4/5 on the left). Sensations of both lower limbs were reduced from L4 downwards. Deep tendon reflexes of the knee and the ankle joints were absent. Plantar reflex was down-going bilaterally.

**Figure 1:**
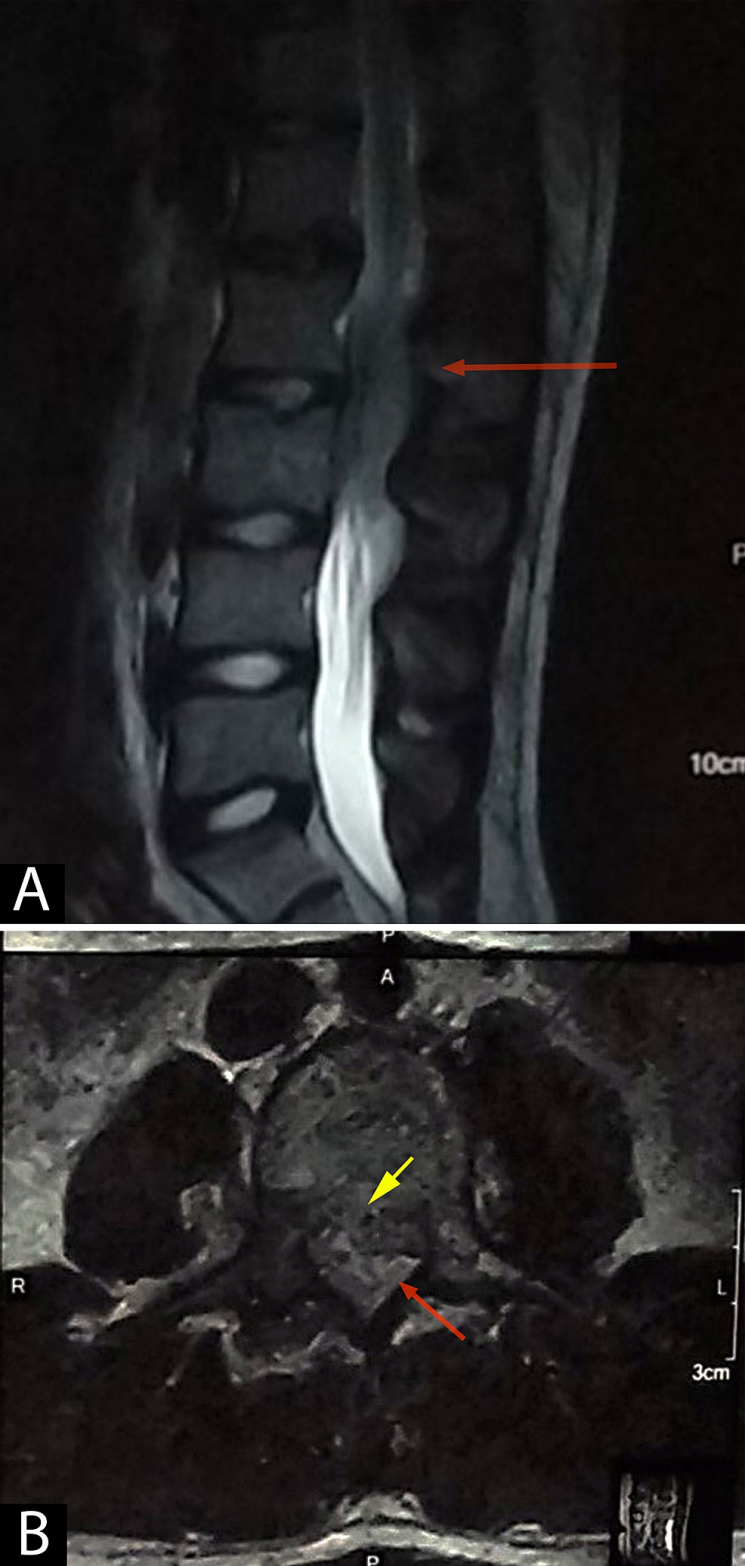
T2 sagittal (A) and axial (B) images showing a heterogeneous iso- to hyper-intense lesion extending from the L2 to L3 vertebral level, causing severe central spinal canal stenosis compressing the thecal sac (red arrows). A suspicious breach in the posterior cortex of the vertebral body is also noted (yellow arrow). Disc spaces between the L2 and L3 vertebral body appear intact. Prevertebral regions appear normal.

A provisional diagnosis of traumatic prolapsed intervertebral disc (with differentials of vertebral fracture, space-occupying lesion, and epidural abscess) was made, and an X-ray of the lumbosacral spine was performed, which showed loss of lumbar lordosis and no bony lesion. Magnetic resonance imaging (MRI) of the spine revealed posterior cortical erosion accompanied by epidural lesion, which was iso-intense on T1 and iso- to hyper-intense on T2, with severe spinal stenosis at L2-L3 vertebrae level (Figure 1).

**Figure 2:**
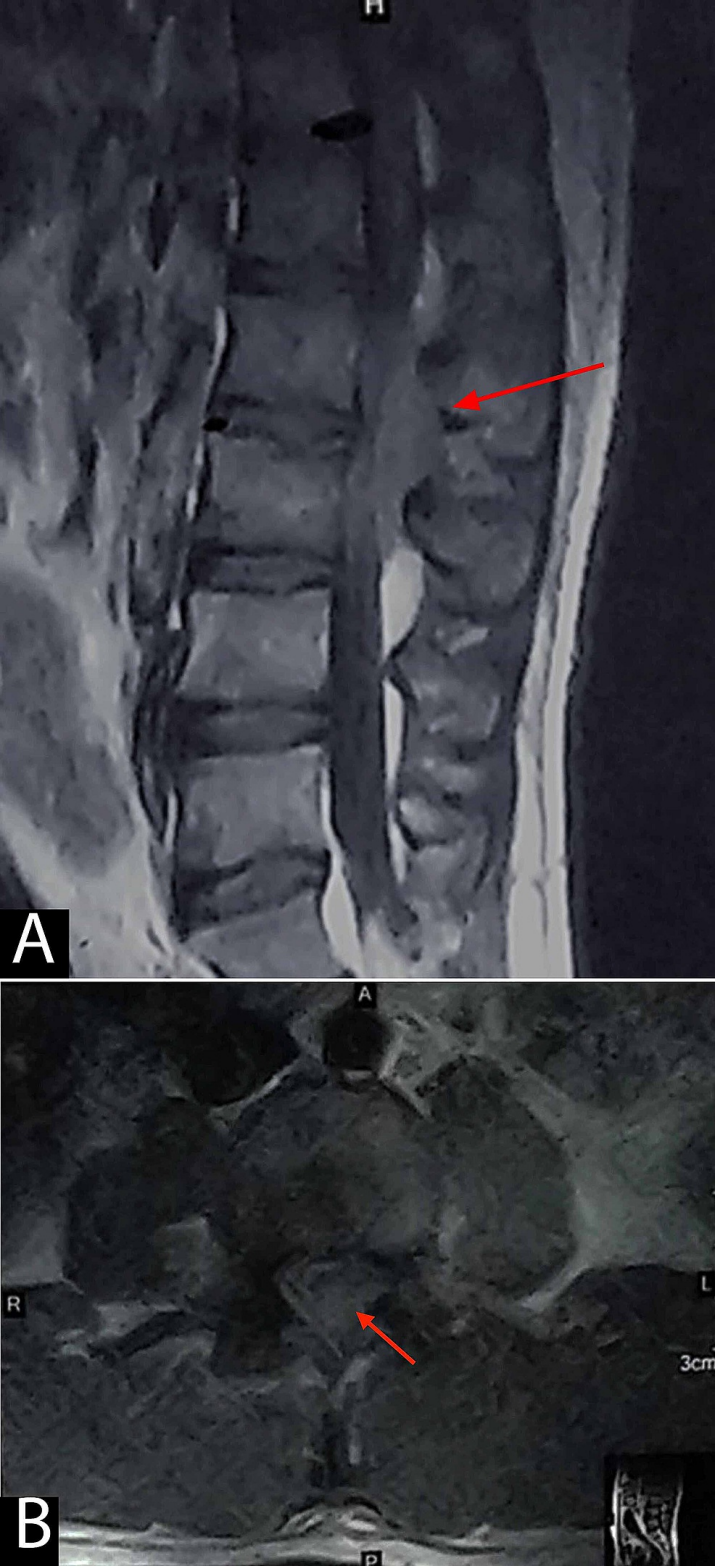
Post-contrast/gadolinium sagittal (A) and axial (B) images showing heterogeneous enhancement of the lesion (red arrows) predominantly occupying the posterior extradural region at the L2-L3 vertebral body levels and severely compressing the thecal sac. However, no abnormal enhancement is seen within the vertebral bodies.

Gadolinium-enhanced MRI showed similar findings with a heterogeneously enhancing lesion extending from L2 to L3, suggestive of an extradural tumor over L2-L3 vertebrae (Figure 2).

Chest X-ray, complete blood count (CBC), liver and renal function tests, peripheral blood smear, lactate dehydrogenase (LDH), uric acid, and urinalysis were normal. However, erythrocytic sedimentation rate (ESR) was 60 mm/hr (normal range: 0-12 mm/hr) and CRP was 205.5 mg/L (normal range: 0-6 mg/L).

**Figure 3:**
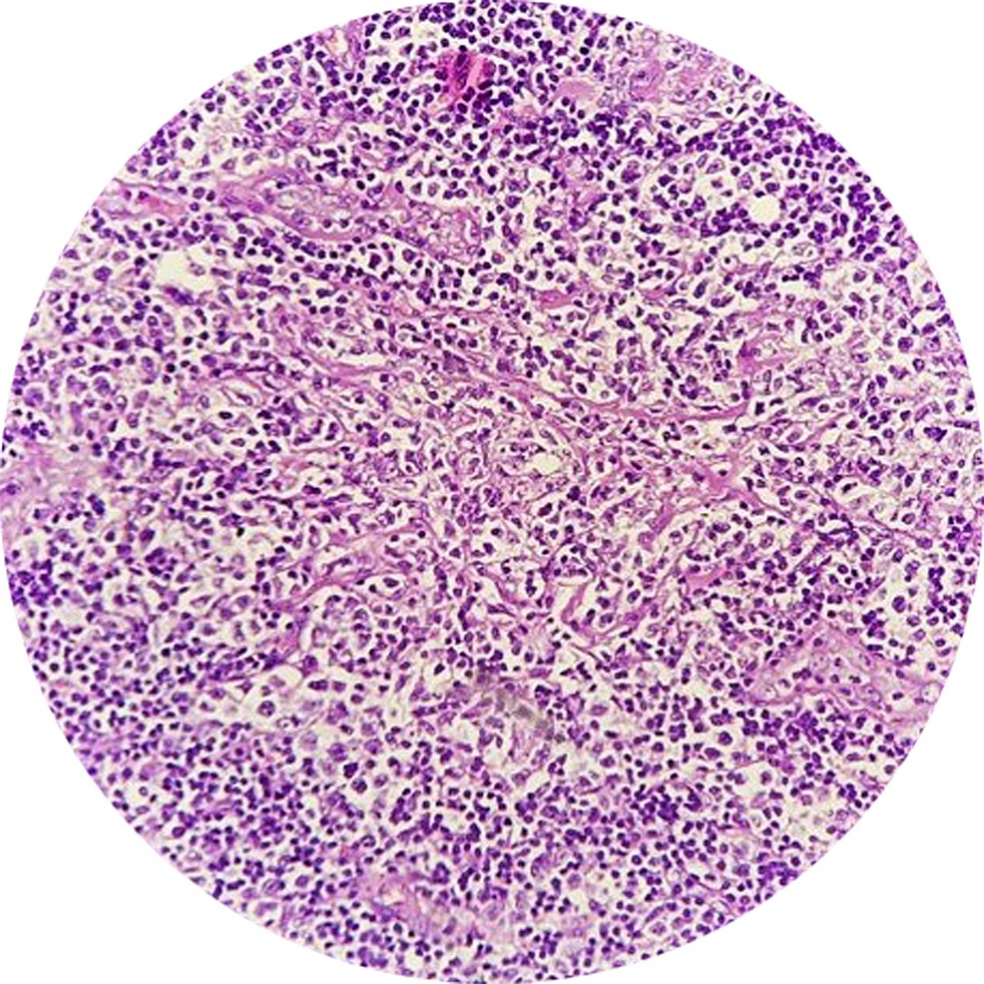
Histopathology (hematoxylin and eosin, x100) revealing diffuse proliferation of lymphoid cells, cleaved lymphocytes with prominent nucleoli, with few blast cells, increase in mitosis, and cells resembling Reed-Sternberg cells.

Decompression (laminectomy) and subtotal excision biopsy of the lesion were performed through the posterior midline approach. Intraoperative findings revealed a grey-colored, circumferential lesion that was soft to firm in consistency and was loosely adhered to the dura. A 4 × 4 cm of the mass (in piecemeal) was sent for histopathology, which showed IMCL (Figure 3).

Immunohistochemistry reported it as CD3^+^, CD5^+^, CD10^-^, CD20^+^, CD23^+^, CD30^-^, CD99^+^, BCL-2^+^, cyclin D1^+^, Ki-67 (<10% positive), and PAX-5^+^.

The postoperative period was uneventful. His radicular pain in both lower limbs improved immediately following surgery. No other lesion was identified on staging evaluation. Contrast-enhanced computed tomography scan (CECT) of the chest, abdomen, and pelvis excluded any accompanying abnormalities. Bone marrow aspiration and biopsy were negative for malignancy. His weakness improved gradually with physiotherapy to 5/5 in all muscle groups in both lower limbs by the time of discharge. Postoperative MRI showed some residual mass at the L2-L3 level. He was comfortably walking when we referred him on the 22nd postoperative day to a cancer center for further treatment (chemotherapy and/or radiotherapy).

At three months follow-up, he was asymptomatic with complete neurological recovery. He is a migrant worker in India. On video-conferencing for a one-year follow-up, he was doing his routine job. He was under follow-up with an oncologist and planned for radiotherapy in a tertiary care center (New Delhi, India). The oncologist has followed the watch-and-wait policy as acceptable management since it was an indolent-type MCL.

## Discussion

MCL is a variant of intermediate lymphocytic lymphoma, a distinctive type of NHL with unique clinical, pathological, and biological features [3]. It can affect the central nervous system either primarily or as a secondary spread. In histology, classical MCL may show a vaguely nodular, diffuse, or mantle zone growth pattern where the tumor cells surround preserved germinal centers as expanded mantle zones. The tumor cells are usually small- to medium-sized with irregular nuclear contours, resembling centrocytes. The immunophenotype of MCL is usually positive for CD5, CD19, and CD20, and negative for CD10 and BCL-6 [10]. Karyotyping can show chromosomal translocation t(11;14)(q13;q32) as a genetic characteristic of MCL, which leads to the aberrant overexpression of cyclin D1, considered to be the hallmark of MCL [11].

Primary spinal epidural lymphoma is rare and accounts for 0.1%-6.5% of all lymphomas and 9% of all spinal epidural tumors [12]. Mantle cell tumors account for 5 % of all NHLs [10]. Men are more affected than women (2:1). The median age is 60 years. MCL is typically diagnosed in patients at an advanced stage of the disease (Ann Arbor stage III/IV). Patient’s presentation would be generalized lymphadenopathy with involvement of the spleen, liver, and bone marrow. Mostly MCL is nodal in origin; the extra-nodal form is rare. The most frequently involved extra-nodal sites are the gastrointestinal tract, Waldeyer's ring, and nasopharynx [13].

Aggressiveness of MCL is determined by over- or under-expression of many genes such as *TP53* and *SOXII* [11], which can help in management planning. According to MIPI, there are three distinct groups. If incorporated with Ki67, the validity of MIPI will be more [7]. Ki67 is a protein expressed during cell replication and its level corresponds with aggressiveness of the tumor. Determann et al. using cut-off values of Ki67 positivity as <10%, 10-30%, and >30% demonstrated three-year overall survival (OS) of 93%, 74%, and 66%, respectively [14].

In the past years, MCL had a poor prognosis, with a five-year OS rate of only 27%. However, introduction of novel drugs and therapeutic options have shown significant improvements in treatment outcome, with a current five-year survival rate of 50%-75% [15].

Clinicians treating MCL have recognized a subset of patients with MCL who have a slow (indolent) disease course with significantly longer survival (>7-10 years). Indolent lymphoma is a slow-growing tumor compared to other aggressive lymphomas. Indolent lymphomas are less dangerous if left untreated; however, they are more difficult to cure because the lower proliferation of cells makes it less susceptible to chemotherapy. For indolent lymphomas, where cure is not an option, the most important treatment may be to avoid overtreatment, *primum non nocere* (above all else do no harm). Nonetheless, nonaggressive, indolent lymphomas can transform over time into aggressive lymphomas [16,17].

Our case presented with typical symptoms [12] of back pain for six weeks followed by bilateral lower limb radicular pain and neurological deficits for nine days with intact bowel and bladder habits and absence of B symptoms (fever, night sweats, and weight loss). Laminectomy and subtotal excisional biopsy were performed as recommended [12]. Positive staining of PAX-5 and CD20, expression of cyclin D1, and less expression of Ki-67 suggest B-cell NHL, MCL type, and indolent course of the disease, respectively.

Unlike Schwechheimer et al.'s and Barnard et al.'s reported cases of spinal epidural MCL, in which the patients were over 70 years of age and had other co-morbidities, our patient was a young, healthy adult with no co-morbidity [8,9]. Both the cases reported by Schwechheimer et al. had lesions in the mid-thoracic region and were treated with surgery alone, and had poor outcomes because of other complications [8]. In the second reported case, the lesion was in the lumbosacral region for which laminectomy and partial resection of the tumor were performed followed by local radiotherapy but developed recurrence in cheek and mediastinum after seven months of treatment, which was further treated with chemotherapy [9]. These reports suggest that all the cases had an aggressive course of the disease. However, a case with bony MCL of thoracic (T10) and lumbar (L1) vertebrae was treated by kyphoplasty and biopsy, followed by eight cycles of combination chemotherapy, and remained in good clinical condition at one-year follow-up except complaining of slight back pain [18].

Our case had an excellent neurological improvement in the lower limbs immediately after the surgery. At three months’ follow-up, he had full neurological recovery. The patient was asymptomatic and has resumed his job in India. Since it was an indolent type of MCL, the oncologist has followed the watch-and-wait policy. It is an acceptable management strategy in selected clinically well patients. The treatment was deferred since the time to treatment did not predict OS, and the median OS of the observation group was statistically longer than that of the early treatment group [16].

We agree with Tsukada et al. that functional recovery of spinal cord compression due to primary spinal epidural lymphoma is relatively better than metastatic carcinoma [19], and early diagnosis and surgery improve functional outcomes [20]. Indolent course of the disease and complete post-treatment neurologic improvement are the most significant favorable prognostic indicators of OS.

## Conclusions

Primary spinal epidural IMCL is an extremely rare tumor due to which its diagnosis is challenging. A high degree of suspicion along with a team approach (spine surgeons, radiotherapists, oncologists, and pathologists) is vital for its diagnosis and management. The young age of the patient without co-morbidity, early diagnosis, and treatment are the keys to a favorable outcome.
